# Increased expression of cysteine cathepsins in ovarian tissue from chickens with ovarian cancer

**DOI:** 10.1186/1477-7827-8-100

**Published:** 2010-08-21

**Authors:** Suzie E Ahn, Jin Won Choi, Deivendran Rengaraj, Hee Won Seo, Whasun Lim, Jae Yong Han, Gwonhwa Song

**Affiliations:** 1 WCU Biomodulation Major, Department of Agricultural Biotechnology, Seoul National University, 599 Gwanak-ro, Gwanak-gu, Seoul 151-921, Korea

## Abstract

**Background:**

Cysteine cathepsins (CTSs) are involved in the degradation and remodeling of the extracellular matrix and are associated with cell transformation, differentiation, motility, and adhesion. These functions are also related to cancer cell invasion and metastasis. Chickens spontaneously develop epithelial ovarian cancer and are therefore a good animal model for human ovarian cancer. However, no studies have investigated the expression of CTSs in chickens with ovarian cancer.

**Methods:**

Cancerous (n = 5) and normal (n = 3) ovaries were collected from 2-to 3-year-old hens, and ovarian tissue samples were collected for study. Ovarian cancers were evaluated with hematoxylin and eosin staining. Reverse transcriptase and quantitative PCR analyses, in situ hybridization analysis were performed to examine the mRNA expression pattern of three CTSs in detail, and protein expression of CTSB was evaluated.

**Results:**

The CTSB, CTSC, and CTSS genes were highly expressed in cancerous chicken ovaries. Messenger RNAs for the three CTSs were localized to a nodule area, a major characteristic of cancerous ovaries, but the three CTSs showed no specific localization in normal ovaries. Immunoreactive CTSB protein was present in the nodule area of cancerous ovaries.

**Conclusion:**

Our results suggest that CTSB, CTSC, and CTSS have important functions in the development of epithelial ovarian cancer.

## Background

Ovarian cancer has the highest mortality rate of all gynecological cancers and is the fifth leading cause of death among women [1]. About 90% of human ovarian cancers are thought to originate from the ovarian surface epithelium [2]. The rate of epithelial ovarian cancer is high because incessant ovulation causes genomic damage to the ovarian surface epithelium, increasing the possibility of gene mutations [3,4]. The lack of an appropriate animal model has prevented mechanistic studies of ovarian cancer [2].

Chickens ovulate almost every day, whereas women ovulate only once a month. Given the prevalent hypothesis that the cause of ovarian cancer is incessant ovulation [5], chickens that spontaneously develop epithelial ovarian cancer may be a good animal model for researching the mechanisms responsible for human ovarian cancer [6,7]. Furthermore, CA125, a well-known marker for human ovarian cancer, is expressed in chicken ovarian cancer cells, but not in normal ovarian cells [6,8,9].

There are 11 cysteine cathepsins in human (CTSB, -C, -F, -H, -K, -L, -O, -S, -V, -W, and -X/Z), which all share a conserved active site formed by cysteine and histidine residues [10]. The CTSs have functions in not only regulation of intracellular protein metabolism [11] but also bone resorption [12] and antigen presentation [13]. In addition, CTSs are involved in the degradation and remodeling of extracellular matrix and are associated with cell transformation, differentiation, motility, and adhesion [14]. These functions are also related to cancer cell invasion and metastasis [15,16]. CTSB is a marker for ovarian cancer prognosis [17] and may contribute to the invasion of ovarian cancer cells [18]. Nevertheless, no studies have investigated the expression of CTSs in chickens with ovarian cancer.

The purpose of this study is to confirm that the expression pattern of CTSs in human is replicated to some extent in the chicken. Therefore, the expression of all known CTSs was examined in normal and cancerous ovaries from chickens, and *in situ *hybridization was used to determine the cell-specific localization of CTSs differentially expressed between normal and cancerous ovaries.

## Methods

### Animals

The care and experimental use of White Leghorn (WL) chickens was approved by the Institute of Laboratory Animal Resources, Seoul National University (SNU-070823-5). The WL chickens were maintained in a standard management program at the University Animal Farm, Seoul National University, Korea. The procedures for animal management, reproduction, and embryo manipulation followed standard operating protocols used in our laboratory.

### Tissue samples

Cancerous (n = 5) and normal (n = 3) ovaries were collected from 2-to 3-year-old WL hens, and ovarian tissue samples were collected for study. We have examined the tumor stage in five chickens with cancerous ovaries according to characteristic features of chicken ovarian cancer previously reported [19]. In three hens, ovarian tumor seeding had metastasized to gastrointestinal tract and superficial surface in liver, and profuse ascites were detected in abdominal cavity. In the other two hens, the tumors had metastasized to distant organs with profuse ascites such as liver parenchyma, lung, gastrointestinal tract and oviduct. Therefore, former three and later two tumors were classified to the stage III and stage IV of chicken ovarian cancer, respectively. Subsets of these samples were frozen or paraffin-embedded for further analyses. Paraffin-embedded tissues were sectioned at 5 μm and stained with hematoxylin and eosin (H&E). Epithelial ovarian cancers in chicken were classified based on the cellular subtypes and patterns of cellular differentiation with reference to ovarian malignant tumor types in humans. In this study, three types including serous (n = 2), endometrioid (n = 2), and clear cell (n = 1) were observed in chicken ovarian tumors.

### RT-PCR analysis

Total RNA was extracted from frozen tissues by Trizol reagent (Invitrogen, Carlsbad, CA), and cDNAs were synthesized using AccuPower^® ^RT PreMix (Bioneer, Daejeon, Korea). The cDNA was serially diluted 10-fold and quantitatively equalized for PCR amplification using specific primer sets (Table 1). The PCR amplification was performed as follows: 1) 95°C for 3 min; 2) 95°C for 20 s, 60°C for 40 s, and 72°C for 1 min for 30 cycles (*CTSS *and *GAPDH*), 35 cycles (*CTSB, CTSC, CTSH, CTSK, CTSL*, and *CTSZ*), or 40 cycles (*CTSO*); and 3) 72°C for 5 min. The PCR products were analyzed using 1% agarose gels with ethidium bromide.

**Table 1:** Primers used for RT-PCR.

Gene	Sequence (5'→3'):	GenBank	Product Size (bp)
	Forward and Reverse	**Accession No**.	
*CTSB*	AGGGCACAACTTCCACAACA GCGAGTAGCCAGGTTCACAG	NM_205371.1	524
*CTSC*	AAAGCCTGCCCCTCTAACAC AGCCTACCAGCAAGACAGCA	XM_417207.2	590
*CTSH*	GGGGCTTTTTAGTGGCTCTG GAAGTCGCTCGTCACCTCAA	XM_001232764.1#1	560
*CTSK*	GAGGAGGTGGTGAGGACGAT AGAACTGGAAGGAGGGCAGA	NM_204971.1	526
*CTSL*	CCTGATTTGGACAGCCACTG AGCCTTGATTTCCTTCTGGG	NM_001168009.1	475
*CTSO*	AGTGCCAAAGGGAGAGGAAA CAAAGGACCCCAGTCAACAA	NM_001031129.1	427
*CTSS*	GCACCCTCAACGAGAAGGA GCACAGAGAAAATCACCCCC	NM_001031345.1	433
*CTSZ*	GTCAACTACGCCAGCACCAC CATTTTCTACACCCCAGCCA	XM_417483.2	538
*GAPDH*	CACAGCCACACAGAAGACGG CCATCAAGTCCACAACACGG	NM_204305	443

### Quantitative RT-PCR analysis

Quantitative RT-PCR was performed using SYBR Green (Sigma, St. Louis, MO) and a StepOnePlus Real-Time PCR System (Applied Biosystems, Foster City, CA). Relative quantification of gene expression was calculated using the formula 2^-ΔΔCt ^, where ΔΔCt = (Ct_target gene _- Ct*_GAPDH_*)_cancerous tissue _- (Ct_target gene _- Ct*_GAPDH_*)_normal tissue_. The information for the primer sets is provided in Table 2.

**Table 2:** Primers used for quantitative RT-PCR.

Gene	Sequence (5'→3'):	GenBank	Product Size (bp)
	Forward and Reverse	**Accession No**.	
*CTSB*	GCACTACGGCATCACATCCT AACCTGCTCCCCTGACACAT	NM_205371.1	157
*CTSC*	CTGGAGAAATGTGAATGGCG CTGGGGACTGAAGACTGGCT	XM_417207.2	151
*CTSS*	TGCCACGTGCTCCAAGTATG CGTGGTTCACCTCCTGTGTG	NM_001031345.1	173
*GAPDH*	ACACAGAAGACGGTGGATGG GGCAGGTCAGGTCAACAACA	NM_204305	193

### *In situ *hybridization analysis

The expression of selected genes was examined using *in situ *hybridization as previously described [20]. For hybridization probes, PCR products were generated from ovarian cancer cDNA with the primers used for RT-PCR analysis. The products were gel-extracted and cloned into pGEM-T Easy Vector (Promega). All plasmids were sequenced using T7 and SP6 primers to certain the genes as expected. After verification of the sequences, a DIG-labeled RNA probe was prepared using a DIG RNA labeling kit (Roche Applied Science, Indianapolis, IN). Frozen sections (10 μm) were mounted on slides pretreated with 3-aminopropyltriethoxysilane (APES, Sigma), dried on a 50°C slide warmer, and fixed in 4% paraformaldehyde in phosphate-buffered saline (PBS). The sections were treated with 1% Triton X-100 in PBS for 20 min and washed three times in PBS. The sections were incubated in a prehybridization mixture containing 50% formamide and 5 × standard saline citrate (SSC) for 15 min at room temperature. After prehybridization, the sections were incubated with a hybridization mixture containing 50% formamide, 5 × SSC, 10% dextran sulfate sodium salt, 0.02% bovine serum albumin, 250 μg/ml yeast tRNA, and denatured DIG-labeled cRNA probe for 18 h at 55°C in a humidified chamber. The sections were washed for stringency in a series of solutions containing formamide and SSC. After blocking with a 1% blocking reagent (Roche), the sections were incubated overnight with sheep anti-DIG antibody conjugated to alkaline phosphatase (Roche). The signal was visualized by exposure to a solution containing 0.4 mM 5-bromo-4-chloro-3-indolyl phosphate, 0.4 mM nitroblue tetrazolium, and 2 mM levamisole (Sigma). All sections were counterstained with 1% (w/v) methyl green (Sigma), and photographs were taken using a Zeiss Axiophot light microscope equipped with an Axiocam HRc camera (Carl Zeiss).

### Immunohistochemistry

The candidate hens with either normal or cancerous ovaries were sacrificed, and their ovaries were collected and fixed in 4% paraformaldehyde. The tissues were embedded in paraffin and sectioned at 5 μm on APES-treated (silanized) slides. The sections were then deparaffinized in xylene and rehydrated to water through a graded series of alcohol. After antigen retrieval by boiling in a citrate buffer (10 mM), the sections were incubated with either mouse anti-PCNA IgG (monoclonal antibody raised against recombinant rat PCNA, Santa Cruz Biotechnology, Santa Cruz, CA), mouse anti-vimentin IgG (monoclonal antibody raised against vimentin purified form bovine lens, Millipore, Billerica, MA), mouse anti-ERBB2 IgG (monoclonal antibody raised against a synthetic peptide from the C-terminus of human ERBB2 protein, Thermo Fisher Scientific, Waltham, MA), or rabbit anti-CTSB IgG (polyclonal antibody raised against recombinant rat procathepsin B, Millipore). Mouse and rabbit IgG were used as negative controls. All antibodies were used at 2 μg/ml in PBS containing 1% BSA. The slides were then treated with an avidin-biotin-peroxidase complex according to the manufacturer's instructions (Vector Laboratories, Burlingame, CA) and visualized using diaminobenzidine tetrahydrochloride (Sigma) as a color substrate. After visualization, the sections were coverslipped using Permount (Fisher Scientific, Pittsburgh, PA).

### Statistical analyses

All statistical analyses were performed using Student's t test using the SAS program (SAS Institute, Cary, NC, USA). Differences were considered significant at a value of *P *< 0.05.

## Results

### Pathological characteristics of chicken ovarian cancer

**Figure 1:**
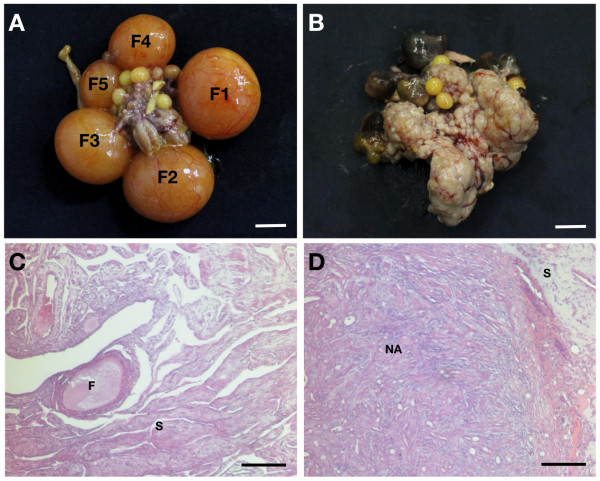
**Pathological characteristics of normal and cancerous chicken ovaries**. Follicles are hierarchically arranged by stage of development (F1-F5) in the normal ovary (A). The cancerous ovary has surface tumor lesions and atretic follicles (B). Hematoxylin and eosin staining of normal (C) and cancerous (D) ovaries from hens. F, follicle; S, stroma; NA, nodule area. *Scale bar = 1 cm *(A and B) or *100 μm *(C and D).

Cancerous ovaries from chickens differed morphologically from normal ovaries. The normal chicken ovary contained large yellow follicles that were hierarchically arranged by stage (Fig. 1A). However, the cancerous ovary was more solid and possessed surface tumor lesions and atretic follicles, indicating abnormal ovarian function (Fig. 1B). Further histological analysis after H&E staining of normal and cancerous ovaries revealed that normal ovaries contained follicles surrounded by connective tissue (Fig. 1C), whereas cancerous ovaries consisted primarily of nodule structures in the solid portion of the ovary (Fig. 1D). These morphological differences between normal and cancerous ovaries were very similar to those reported previously [21-24].

### Immunohistochemical characterization of chicken ovarian cancer

**Figure 2:**
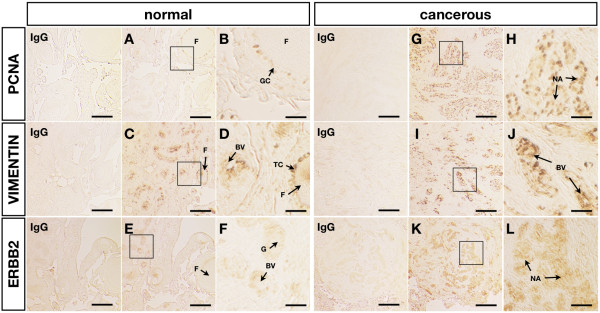
**Immunohistochemical analysis of normal and cancerous chicken ovaries**. PCNA (A, B, G, and H), vimentin (C, D, I, and J), and ERBB2 (E, F, K, and L). In the IgG control, normal mouse IgG was substituted for antibody against each specific antibody. F, follicle; S, stroma; BV, blood vessel; NA, nodule area; G, gland; GC, granulosa cell; TC, theca cell. *Scale bar = 100 μm *(A, C, E, G, I, and K) or *25 μm *(B, D, F, H, J, and L).

We performed immunohistochemistry to further characterize cancerous chicken ovaries based on reports that proliferating cell nuclear antigen (PCNA), vimentin, and ERBB2 proteins were detected in cancerous ovaries of chickens [8,25,26]. In the present study, immunoreactive PCNA was detected in granulosa cells surrounding follicles in normal ovaries (Fig. 2A and 2B), and vimentin was localized to the cells surrounding the granulosa cell layer and blood vessels (Fig. 2C and 2D). ERBB2 was also weakly expressed in glands and blood vessels (Fig. 2E and 2F). In cancerous ovaries, PCNA protein was predominantly detected in the nucleus of cancerous cells in the nodule area (Fig. 2G and 2H). However, vimentin was not expressed in cancerous areas of the ovaries, but was detected in blood vessels (Fig. 2I and 2J). ERBB2 was localized to the cytoplasm in the nodule area of cancerous ovaries (Fig. 2K and 2L).

### Differential expression of CTSB, CTSC, and CTSS in normal and cancerous ovaries

**Figure 3:**
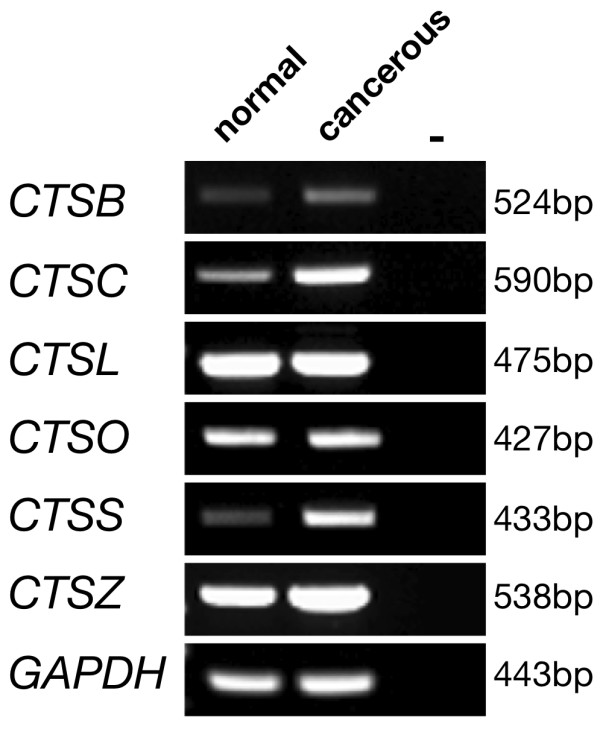
**RT-PCR analysis of normal and cancerous chicken ovaries**.

Based on the morphological and immunohistochemical differences between normal and cancerous hen ovaries, we hypothesized that expression patterns of the various CTSs related to cancer may differ between normal and cancerous tissues. First, the expression of all known CTSs in cancerous chicken ovaries was examined by RT-PCR analysis, and six CTSs were found to be expressed in both normal and cancerous ovaries (Fig. 3). *CTSH *and *CTSK *were not detected by RT-PCR (data not shown).

**Figure 4:**
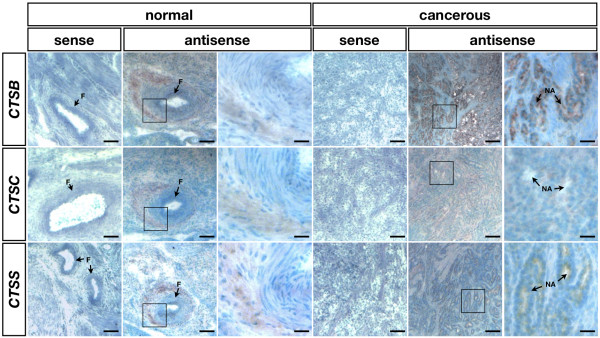
***In situ *hybridization analysis for cell-specific expression**. Frozen sections of normal and cancerous hen ovaries were subjected to *in situ *hybridization analysis against sense and/or antisense probes of *CTSB, CTSC*, and *CTSS*. There was no mRNA expression detected in the normal and cancerous ovaries hybridized with sense probes of *CTSB, CTSC*, or *CTSS*. A low level cell-specific localization of *CTSB, CTSC*, or *CTSS *mRNA was detected in normal ovaries. *CTSB *mRNA was strongly expressed in the nodule area of cancerous hen ovaries. *CTSC *and *CTSS *mRNAs were also expressed slightly higher level in the gland-like area of cancerous ovaries than that of normal ovaries. F, follicle; NA, nodule area. *Scale bar = 100 μm *(columns 1-2 and 4-5) *and 25 μm *(columns 3 and 6).

**Figure 5:**
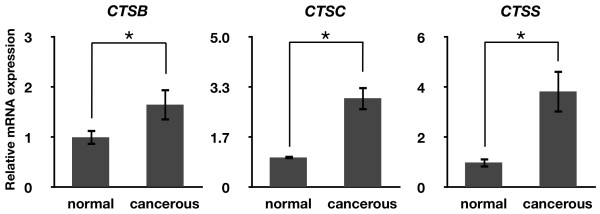
**Quantitative RT-PCR analysis in normal and cancerous chicken ovaries**. Relative expression of *CTSB, CTSC*, and *CTSS *mRNAs in normal and cancerous chicken ovaries, showing that the expression of *CTSB, CTSC*, and *CTSS *mRNAs was greater in cancerous ovaries (mean ± SEM; *P *< 0.05).

*In situ *hybridization analysis was used to determine cell-specific localization of *CTSB*, *CTSC*, and *CTSS *mRNAs (Fig. 4). *CTSB *was expressed at a low level around follicles in the normal ovaries, but there was strong expression of *CTSB *mRNA in the nodule area in cancerous ovaries. Similarly, there was localization of *CTSC *mRNA around follicles in normal ovaries, whereas *CTSC *mRNA was weakly expressed in the nodule area of cancerous ovaries. Although there was also localization of *CTSS *mRNA around follicles in normal ovaries, *CTSS *mRNA was abundant in the nodule area of cancerous ovaries. Further analysis using quantitative RT-PCR indicated that mRNA expression levels for *CTSB*, *CTSC*, and *CTSS *were higher in cancerous ovaries (*P *< 0.05, Fig. 5).

### Localization of immunoreactive CTSB protein in cancerous ovaries

**Figure 6:**
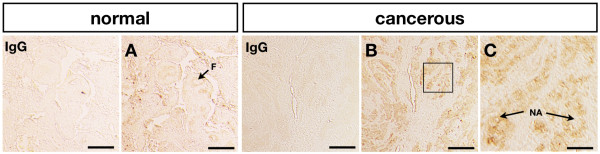
**Localization of immunoreactive CTSB protein in normal and cancerous chicken ovaries**. CTSB protein was detected around follicles in normal hen ovaries (A). In cancerous hen ovaries, CTSB protein was localized to the nodule area of cancer cells (B and C). In the IgG control, normal mouse IgG was substituted for antibody against each specific antibody. F, follicle; NA, nodule area. *Scale bar = 100 μm *(B) and *Scale bar = 25 μm *(C).

We further confirmed the localization of immunoreactive CTSB protein by immunohistochemistry and found regions of staining around follicles in normal ovaries (Fig. 6A); however, CTSB protein was detected in the nodule area in cancerous ovaries (Fig. 6B and 6C) which was consistent with the differential expression of normal and cancerous ovarian *CTSB *mRNA (Fig. 4 and 5). Moreover, CTSB was identified in the cytoplasm of tumor cells (Fig. 6B) showing different pattern from staining with anti-PCNA antibody in which PCNA was identified in nucleus (Fig. 2G and 2H).

## Discussion

The mechanisms responsible for the development of ovarian cancer are not fully understood owing to the lack of a suitable ovarian cancer animal model [2]. However, the laying hen has received attention as a model for ovarian cancer research because of significant similarities between ovarian cancers of hens and women. Ovarian adenocarcinomas originate predominantly from the ovarian epithelium and are associated with incessant ovulation in both species [3,5,27]. The laying hen model strongly supports the incessant ovulation theory, as hens ovulate almost every day and exhibit high rates of spontaneous development of ovarian adenocarcinoma [6,27]. Also, several anti-tumor antibody antigens (e.g., cytokeratin, PCNA, COX-1, COX-2, CEA, AE1/AE3, EGER, ERBB2, Lewis Y, SELENBP1, p53 and Tag 72) that are commonly used as markers for human ovarian cancer are also detected in ovarian carcinomas of hens [6,8,21,23,24,26,28,29].

Proteases, which catalyze the cleavage of peptide bonds in proteins, can be divided into five categories: metalloproteases, cysteine proteases, serine proteases, aspartic proteases, and threonine proteases [30]. CTSs are a family of cysteine proteases that function primarily in protein degradation in the lysosomes of the majority of cell types [11]. However, specific CTSs are often upregulated in various cancers [31]. CTSs are expressed at the cell surface of cancer cells and secreted into the extracellular space, where they degrade ECM components [15,32]. This extracellular proteolytic activity allows cancer cells to invade surrounding tissue, blood, and lymph vessels and to metastasize to tissues at distant sites [33]. These important roles of CTSs in cancer development encouraged us to examine the expression of CTSs in the chicken ovarian cancer model. Specific expression of *CTSB, CTSC*, and *CTSS *was clearly observed in cancerous ovaries of hens.

Among the CTSs, CTSB has been investigated most intensively and appears to play a role in cancer based on its increased expression in various human cancers [34-36]. A role of CTSB in tumor cell invasion was suggested by the increased invasiveness of cells overexpressing CTSB [37] and by decreased invasion in the presence of specific inhibitors of CTSB [38]. Women with ovarian cancer have higher levels of CTSB in their sera [39], and CTSB is present in ascites and cyst fluid of patients with ovarian cancer [40,41]. Moreover, immunohistochemical analysis has shown that CTSB is evident in the cytoplasm of tumor cells in human ovarian cancer [18,42]. Similarly, the results of the present study indicate increased expression of CTSB in cancerous, but not normal, chicken ovaries. This suggests that the role for CSTB in tumor invasion in chickens may be similar to that in human ovarian cancer.

In addition to CTSB, other CTSs have been proposed as participants in the angiogenesis and invasion of tumor cells. For example, *Ctss*-deficient mice displayed defective microvessel development during wound repair, owing to the reduced ability of endothelial cells to invade the ECM [43]. In a murine model of sporadic pancreatic carcinogenesis, null mutant *Ctsb *and *Ctss *mice exhibit decreased tumor invasion and angiogenesis [44,45]. Another study demonstrated that both CTSB and CTSS are upregulated in the transition from normal to angiogenic islets and that CTSC is expressed concomitantly with the development of angiogenic islets in mouse pancreatic islet tumors [46]. In the present study, the expression of *CTSS *and *CTSC *was also detected in cancerous ovaries of hens, suggesting that CTSS and CTSC may also play roles in the angiogenesis and invasion of tumor cells.

## Conclusions

The results of the present study demonstrate that *CTSB*, *CTSC*, and *CTSS *are upregulated in cancerous ovaries of chickens, suggesting that CTSB, CTSC, and CTSS have potentially important functions in the development of ovarian cancer in chickens. Our study, therefore, provides a basis for the development of the hen as an animal model for the study of human ovarian cancer and for the discovery of the mechanisms responsible for the development of ovarian cancer.

## Competing interests

The authors declare that they have no competing interests.

## Authors' contributions

GS coordinated all steps of the study. SEA and JWC carried out all the experimental procedures and data. DR carried out the *in situ *hybridization analysis. HWS and LW examined and selected the images. JYH interpreted and analyzed the results. All authors participated in the design and writing of this study. All authors read and approved the final manuscript.
